# Researching multimorbidity in hospital: can we deliver on the promise of health informatics?

**DOI:** 10.1007/s41999-023-00753-6

**Published:** 2023-05-25

**Authors:** Miles D. Witham, Rachel Cooper, Paolo Missier, Sian M. Robinson, Elizabeth Sapey, Avan A. Sayer

**Affiliations:** 1 AGE Research Group, Translational and Clinical Research Institute, Faculty of Medical Sciences, Newcastle University, Newcastle, UK; 2 NIHR Newcastle Biomedical Research Centre, Newcastle University, Newcastle Upon Tyne NHS Foundation Trust and Cumbria, Northumberland, Tyne and Wear NHS Foundation Trust, Biomedical Research Building, Campus for Ageing and Vitality, Newcastle Upon Tyne, NE4 5PL UK; 3 School of Computing, Newcastle University, Newcastle Upon Tyne, UK; 4 Institute of Inflammation and Ageing, University of Birmingham, Birmingham, UK; 5 PIONEER Hub, University of Birmingham, Birmingham, UK

**Keywords:** Multimorbidity, Big data, Hospital, Electronic health care record, Health informatics

Multimorbidity (sometimes referred to as multiple long-term conditions; MLTC) refers to the coexistence of two or more chronic health conditions and has been described as posing one of the greatest challenges to medicine and science in the twenty-first century [1]. There are many conditions that modern medicine cannot cure, and until this changes, conditions will accumulate across the life course with older people facing the highest burden of multimorbidity [2, 3]. The prognosis of people with multimorbidity is considerably worse than prognosis of people with single conditions, especially if the set of conditions a person is living with includes mental health conditions [4]. A higher burden of symptoms and care, a greater chance of functional decline and worse quality of life are all consequences of multimorbidity [5]. These problems are compounded by a plethora of clinical guidelines that focus on the diagnosis and management of single conditions in isolation [6, 7].

This is particularly true for hospital-based care. Many components of hospital care are not designed or equipped to deliver care efficiently and effectively for people living with multimorbidity. Healthcare workers in secondary care typically specialise in single organ-based diseases and may lack the skills to manage conditions affecting other organs or to understand the impact of other conditions on the index condition. Although geriatricians have generalist skills and processes designed to manage MLTC in hospital, the scale of multimorbidity is such that a whole-system approach is needed to improving hospital care across all specialties. Patients with multimorbidity admitted to hospital constitute a distinct and select subset of people living with multimorbidity—they are by definition unwell enough to require hospital admission, and the patterns, mechanisms and prognosis of their multimorbidity may differ from the general population.

Most research into multimorbidity to date has used large primary care or population-level data sets. We therefore lack important information about how patients with multimorbidity present to, or are managed by, secondary care services. Conducting research into multimorbidity in patients admitted to hospital requires overcoming a series of key challenges, some of which are listed in Table 1. Importantly, if we are to fully understand and improve care for people with multimorbidity admitted to hospital, a single-disease silo approach to research is inappropriate. A particular challenge is that conditions interact with each other, in terms of predisposition to disease, disease presentation and prognosis. Multimorbidity can lead to multiple therapeutic interventions and is a key driver of polypharmacy. Polypharmacy in turn magnifies the risk of side effects and of precipitating further medication-induced conditions. Furthermore, shared biological processes may drive the occurrence and progression of several long-term conditions—a focus on individual diseases, rather than on these underlying common mechanisms, means that we may miss opportunities to treat multiple conditions with the same intervention.

**Table 1:** Opportunities and challenges in using routinely collected data captured in electronic hospital records for MLTC research

**Opportunities**
Volume (number of individuals included in datasets)
Variety of data (including prescribing, laboratory data, images and physiological measures)
Generalisability—routine data includes unselected healthcare users
Longitudinal data (repeated, sometimes frequent) contacts over long periods of time
**Challenges**
Lack of interoperability between provider systems or data held in systems inaccessible to research teams
Not all data are in electronic form, and even when present, may be in unstructured formats and difficult to extract
Lack of quality control and challenges with data integration—nonsense values, erroneous values and variation in how users code diagnoses and derive other variables
Missing data—due to variables representing a construct not existing in a dataset and to individual values missing; irregular intervals between visits or contacts
Lack of consistency in how diagnoses are arrived at—overdiagnosis, underdiagnosis and misdiagnosis
Lacks join up to primary care and social care data
Not all outcomes important to patients may be recorded
Data governance arrangements may lead to delays or constrain data sharing

How can big data help to tackle these challenges? The scale and complexity of hospital-based multimorbidity research speaks to the need for both large datasets and granular data. Fortunately, a growing number of hospitals across Europe now possess electronic healthcare records (EHR). Exploiting these sources of routinely collected clinical data provides an unrivalled opportunity to conduct hospital-based multimorbidity research. Doing so is not straightforward, however [8]. First, there is no consensus on what conditions to study, or even on what counts as a long-term condition, although recent consensus initiatives are catalysing progress in this area [9]. Not all diagnoses are equally important in terms of either symptoms, burden of care or prognosis, and what is important to hospitals may not always be what is important to primary care. Second, routine data are complex. Whilst simple coded data (e.g. discharge diagnosis codes) are helpful, the real power of hospital EHR lies in the large amounts of unstructured data (images, videos, text entries, letters and scanned documents), as well as potentially thousands of different structured variables including diagnostic codes, vital signs and other clinical measurements, prescriptions and administrative data. Third, routine hospital data are messy. Missing data are common, and diagnoses may not be made or recorded accurately or consistently [10]. The vigour with which diagnoses are pursued and recorded varies depending on the speciality of the healthcare team, and the more contact an individual has with healthcare services, the more diagnoses they will tend to accumulate—referred to as informed presence bias. Finally, studying hospital EHR in isolation is insufficient to understand trajectories of care before hospital admission and after discharge. Ensuring that secondary care data are linked to primary care data and other relevant data sources including social care data is also important to ensure that the study of multimorbidity in hospitalised patients is not confined to the hospital admission alone, and this is a complex task in terms of governance and data management.

None of this should negate the fact that there are key strengths of using data from EHR. The large size of data sets, ranging from thousands to millions of individuals, provides statistical power to enable a broad range of analytic techniques, from descriptive statistics to machine learning techniques and sophisticated clustering methods. Machine learning may be particularly powerful in making sense of complex pathways of care or in identifying possible common underlying mechanisms from very large phenotypic data sets. Data from EHR also enable inclusion of patient groups who are typically under-represented in research (for instance some ethnic minority groups and people from more deprived backgrounds) and those who cannot easily participate in consented cohort studies—for example those with delirium, dementia, or who are too ill to consent. Using routine data thus provides more representative data with which to study multimorbidity in the hospital setting, and the wide range of available data sources can capture diagnoses, treatment and outcomes beyond those recorded by simple hospital discharge coding.

How then can we get the most out of routine data as a research tool for hospitalised people with multimorbidity? We suggest three areas of focus: interoperability and connectedness, maximising the relevance of data contained in the hospital record, and developing research and clinical teams who can deliver data driven multimorbidity research and care. In terms of interoperability and connectedness, we need to align diagnostic information across different data sets in a consistent way. This is essential if analyses are to be replicable across different settings. Initiatives such as the Observational Medical Outcomes Partnership (OMOP) are starting to gain traction in this area, with funding from the European Health Data and Evidence Network (EHDEN; www.ehden.eu) in place to accelerate the transition to this common data model. Open-source resources such as the CALIBER project (www.caliberresearch.org) are providing algorithms for defining conditions across different data sources from primary and secondary care. An increasing number of initiatives, including initiatives in the UK, Canada and New Zealand, as well as initiatives forming part of the Survey of Health, Ageing and Retirement in Europe (SHARE) [11, 12]) seek to link primary care, secondary care and even social care data. In the UK, the Health Data Research UK acute care data hub (PIONEER), provides a leading exemplar of how multiple data sources relevant to acute care can be brought together and managed under a single governance process with robust data management and confidentiality processes [13], in line with the recommendations of the recent Goldacre review (www.goldacrereview.org).

To maximise the relevance of data contained in the hospital record, we need to improve the breadth and quality of information and find new ways to extract diagnoses as well as other information from unstructured data. Natural Language Processing (NLP) applied to machine-readable text is a key technology for this, but applying NLP to confidential patient data presents governance challenges, and deriving ‘ground truth’ (i.e. gold standard diagnoses) for large numbers of patients to validate NLP algorithms still requires expert human participation. However, there are a growing number of instances where this approach has yielded success, including the ability to track cancer trajectories, mental health diagnoses, and delirium [14]. At present, most of these initiatives are confined to single institutions, but opportunities exist to scale up implementation across multiple centres. Additionally, clinicians caring for older people need to be encouraged to improve the recording of outcomes that are important to patients. Whilst information on length of stay, death and readmission to hospital are usually captured and coded well in EHR, many other important pieces of information are not recorded in a structured or consistent manner. This includes physical function, activities of daily living, quality of life and discharge destination, as well as measures of the experience of, and satisfaction with, healthcare delivery—‘quality of stay’. Patients and carers should have a key role in shaping the development of measures and systems to collect these data at scale.

To develop research and clinical teams who can deliver data-driven multimorbidity research and care will require a range of initiatives. For example, building communities of practice to facilitate sharing of methods, structures and clinical expertise will help to build much-needed capacity in health informatics research. Alongside this, training cadres of individuals who can work across the boundaries of data science and healthcare will be key in enabling this research to be delivered at scale and pace. Importantly, the large salaries that appropriately trained interdisciplinary researchers can command from the private sector both emphasises their value but also poses a challenge to the traditional, relatively low-paid academic sector model of research. Novel posts (for instance clinical informatics or data science clinical fellowships) and new career pathways for public-sector data specialists will both be needed.

Fortunately, progress across these three areas of focus is now accelerating. Multimorbidity and the closely related field of integrated care for chronic conditions has been the focus of major European research investment across five projects in recent years [15]. In the UK, the Medical Research Council (MRC) and the National Institute for Health and Care Research (NIHR) have co-funded a series of strategic programmes on multimorbidity [16] which aim to build communities of practice, interdisciplinary training opportunities and methodological expertise in multimorbidity research. One of these programmes (the ADMISSION collaborative; www.admissioncollab.org) has a specific focus on multimorbidity in hospitalised patients, using routine data from across the acute care pathway to describe the burden of multimorbidity, understand the influence of socio-demographic inequalities, map patient pathways and the lived experience of hospitalised patients with multimorbidity, and understand underpinning mechanisms using genetic epidemiology and phenotyping studies. These findings will provide the foundations for delivering interventions that treat multimorbidity with single therapies, and will enable hospital-based healthcare processes to be re-engineered to better meet the needs of hospitalised patients with multimorbidity. Until it is possible to cure conditions that are currently deemed to be chronic in nature, the need for such improvements in care for people living with multimorbidity will only continue to grow.

Meeting these challenges requires new thinking and novel research techniques. Geriatricians and other healthcare professionals looking after older people are ideally placed to lead such initiatives, although doing so may require a move towards intervention earlier in the life course rather than waiting to manage the consequences of multimorbidity such as frailty and disability. Improving infrastructure (interoperability and connectedness), data (relevance and quality) and developing people, together hold the key to rapid advances in this area. Realising this potential will require close and interdisciplinary cooperation between patients and the public, researchers, clinicians, data scientists and care providers.

## Data Availability

Not applicable.
